# Questions remain about the biolability of dissolved black carbon along the combustion continuum

**DOI:** 10.1038/s41467-021-24477-y

**Published:** 2021-07-13

**Authors:** Sasha Wagner, Alysha I. Coppola, Aron Stubbins, Thorsten Dittmar, Jutta Niggemann, Travis W. Drake, Michael Seidel, Robert G. M. Spencer, Hongyan Bao

**Affiliations:** 1 Department of Earth and Environmental Sciences, Rensselaer Polytechnic Institute, Troy, New York, NY USA; 2 Geological Institute, Department of Earth Sciences, ETH Zürich, Zürich, Switzerland; 3 Department of Marine and Environmental Sciences, Civil and Environmental Engineering, and Chemistry and Chemical Biology, Northeastern University, Boston, MA USA; 4 Institute for Chemistry and Biology of the Marine Environment (ICBM), University of Oldenburg, Oldenburg, Germany; 5 Helmholtz Institute for Functional Marine Biodiversity (HIFMB) at the University of Oldenburg, Oldenburg, Germany; 6 Department of Environmental Systems Science, ETH Zurich, Zurich, Switzerland; 7 Department of Earth, Ocean, and Atmospheric Science, Florida State University, Tallahassee, FL USA; 8 State Key Laboratory of Marine Environmental Science, College of Ocean and Earth Sciences, Xiamen University, Xiamen, China

**Keywords:** Carbon cycle, Marine chemistry

**Arising from** Y. Qi et al. *Nature Communications* 10.1038/s41467-020-18808-8 (2020).

The heating and partial combustion of organic matter produces a continuum of organic compounds ranging from partially altered, biolabile molecules to highly refractory graphite^1^. The apparent biolability of compounds formed at lower charring temperatures (<300 °C)^2^ is akin to the process of humans cooking food to improve digestibility and nutrient absorption. Here, the term biolability refers to carbon fractions with rapid microbial turnover rates (within days to weeks). At high charring temperatures (>450 °C), organic matter can undergo aromatization to form polycondensed and graphitic type structures that resist biodegradation^3,4^. Black carbon (BC) refers to the condensed aromatic fraction of thermally altered biomass that accumulates in carbon reservoirs over thousands of years. This long-lived BC fraction has become synonymous with refractory organic carbon (OC) and has garnered the most attention in recent years because it shows promise in terms of carbon sequestration and mitigation strategies. After formation, the composition of BC cycled in aquatic systems is further modulated by hydrophobicity, which governs the partitioning of BC molecules to the dissolved phase (DBC)^5,6^. Qi et al^7^. adapt this definition and describe BC as a “group of condensed byproduct chemicals,” which is consistent with their use of chemothermal oxidation (CTO) to quantify and characterize the thermally stable, residual fraction of BC that remains after heating. Other analytical approaches, such as the benzenepolycarboxylic acid (BPCA) method, access condensed aromatic fractions of BC by oxidative chemistry paired with radiocarbon dating rather than inferring thermal stability ^8,9^.

Condensed aromatic BC, as measured by the BPCA method, comprises a significant fraction of OC in soils (14%)^10^ and of dissolved OC (DOC) in rivers (2–15%)^11^ and the ocean (1–6%)^12,13^, thus establishing the importance of this refractory carbon pool on a global scale. As BC research has expanded and diversified, so have the methodological approaches used to characterize this thermally altered component of DOC. Radiocarbon (Δ^14^C) and stable carbon (δ^13^C) isotopic measurements are increasingly employed to gain insight into potential sources and environmental residence times of DBC across aquatic systems. A simple calculation suggests the global riverine DBC flux (18 Tg-C per year)^11^ could replace the standing stock of oceanic DBC (14 Pg-C)^12^ in <800 years. However, this estimate is at odds with BPCA-specific Δ^14^C measurements, which indicates the residence time of oceanic DBC is 1–2 orders of magnitude longer^12^. This offset might be explained by petrogenic contributions and/or widespread pre-aging of DBC in soils or other intermediate reservoirs prior to riverine export, but recent research showed riverine DBC to be heterogeneous in age even within the same catchment^14^. Further adding to this discrepancy is the δ^13^C signatures of oceanic BPCAs, which are significantly ^13^C-enriched compared to global rivers and suggests condensed aromatic DBC may have a marine origin that remains unknown^13^. Isotopic probing of DBC has clearly raised more questions than it has answered. Having conducted an impressive, large-scale effort to quantify and isotopically characterize DBC, Qi et al^7^. address this conundrum from the CTO analytical perspective.

Similarities between DBC:DOC ratios determined by CTO^7^ and BPCAs suggest some degree of overlap in DBC captured by the two analytical methods. Isotopic agreement between DBC and DOC shown by Qi et al^7^. suggests that CTO-derived DBC is of similar source and bioreactivity as bulk DOC. Although δ^13^C trends mirror those observed by Wagner et al.^13^, similarities between DBC and DOC Δ^14^C values are in opposition to large isotopic offsets described previously for BPCA-derived DBC^12,14^. Given discrepancies in radiocarbon composition between DBC isolated by CTO and BPCA methods, the measurement of DOC and BC standards^15^ is recommended to allow more informed intercomparisons of CTO and BPCA-derived DBC reactivities and residence times. A comparative isotopic assessment of BC standards via CTO would also alleviate or clarify concerns regarding analytical artifacts associated with the method. For example, the accidental transformation of sugars to BC during the CTO heating step has indicated the technique is prone to false positives^16^. However, Qi et al^7^. did attempt to rule out the possibility of false positives under their CTO methodological conditions, stating that they “did not detect any measurable DBC converted from fresh DOC” that was derived from fresh marsh plants and coastal phytoplankton.

Qi et al^7^. ascribe the aging (i.e., ^14^C-depletion) of DBC along the land-to-ocean continuum to in situ biodegradation of the CTO-derived fraction of condensed aromatic carbon. To test the biolability of DBC, the authors carry out a biodegradation experiment, in which bulk DOC is leached from wood charcoal and exposed to microbial activity simultaneously. The biodegradation observed for charcoal-leached DOC is then used to justify the presumed biolability of CTO-derived DBC in rivers. The approach taken by Qi et al^7^. to investigate DBC biolability leads to three concerns: First, the authors conducted leaching and biodegradation experiments at the same time, which precludes any direct assessment of DBC biolability. Under the described experimental conditions, it is impossible to disentangle leaching versus biodegradation processes. Second, the apparent lack of replication raises questions about the repeatability and scalability of their results. Previous research has demonstrated extreme heterogeneity for different types of charcoal leachates, in terms of both carbon yield and composition^5,6^. Therefore, it is unknown how representative the wood charcoal selected by Qi et al^7^. is of charcoal-derived DOC or DBC entering aquatic systems on larger scales. Third, and perhaps most importantly, Qi et al^7^. conflate the understanding of what is molecularly represented by the DBC window with that of the entire combustion continuum. The former is a subset of the latter (Fig. 1). The full suite of DOC molecules leached by charcoal include materials that have a very different set of physicochemical characteristics and biological reactivities than what has been observed for the condensed aromatic DBC fraction. Charcoal leachates contain an abundance of polar functional groups and low molecular weight organic species that facilitate high microbial turnover rates^17,18^. The fact that dehydrated carbohydrates (anhydrosugars) and other small, soluble molecules are abundant in charcoal leachate does not mean that this fraction of released DOC is DBC. Said another way, these biolabile molecules are indeed part of the thermally altered byproducts of biomass combustion, but not part of the condensed aromatic compound class targeted using BC analytical techniques (e.g., BPCA and CTO methods). In fact, a recent experimental study has shown that the BPCA-derived DBC fraction of charcoal leachates is highly resistant to microbial degradation^4^. Therefore, the authors’ key finding that biodegradation is the likely mechanism for losses of CTO-derived DBC during transit is unfounded. Further research is required to fully assess the biolability of the various pyrogenic subcomponents by measuring losses of CTO-derived, BPCA-derived, and other fractions of DBC directly.

**Fig. 1:**
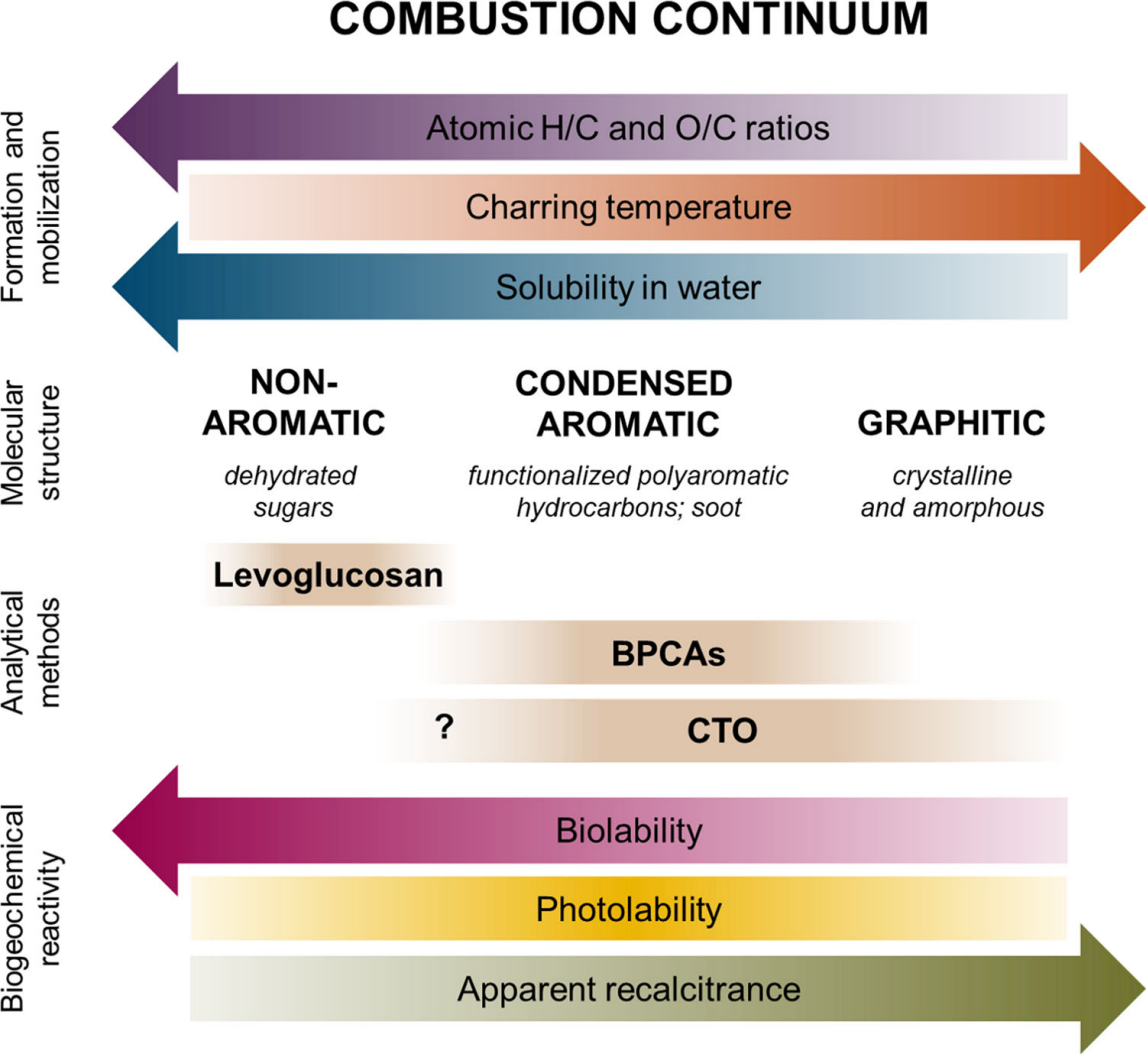
The theoretical combustion continuum. The combustion continuum ranges from mildly thermally altered biomolecules to highly condensed and graphitic structures and describes the formation, composition, and reactivity of different carbon fractions. Arrows indicate the direction of increase. Peak photolability is expected for the condensed aromatic fraction of dissolved black carbon (DBC). The presumed analytical windows for levoglucosan, benzenepolycarboxylic acid (BPCAs), and chemothermal oxidation (CTO) methods are also shown. Data in Qi et al. (2020) suggest CTO captures more than just graphitic DBC, therefore we have expanded the range for this method and included a question mark to denote that further research is needed to understand the breadth of this DBC fraction.

DBC is operationally defined by the method used to quantify and characterize it. When introducing their study, Qi et al^7^. clearly defined DBC as the thermally resistant fraction recovered by the CTO method. However, this definition was not kept consistent when describing the results of the biodegradation experiment, in which “all organic compounds dissolved from the biochar were treated as DBC.” This is an honest statement of an assumption that is too simplistic, which led to what is probably an erroneous overestimate of DBC loss from biodegradation during riverine transit. The combustion continuum describes a complex and heterogenous mixture of all OC (including BC) that is derived from heating, thus its apparent biolability is an emergent property that varies with both composition and environmental conditions (Fig. 1). Published research that investigates the biogeochemical processing of thermally altered organic matter should make this point obvious and clearly define the analytical window through which DBC is being viewed. Although the research community is still working to define the structural edges of each method’s analytical window, we have a good sense of where different methods sit along the combustion continuum (Fig. 1). Since we cannot offer objective structural terms for each segment of the combustion continuum at present, we recommend that authors be explicit in stating the range of chemical structures their chosen method is presumed to capture when describing research findings. Furthermore, if two or more methods are employed in the same study, then unique monikers should be applied (e.g., bulk DOC, DBC_CTO_, DBC_BPCA_) when describing results of carbon-based methods to avoid misleading readers. Our recommendations aim to promote more accurate comparisons of DBC-specific reactivities and the reactivity of thermally altered material more broadly. In doing so, we can bring meaningful biogeochemical context to the environmental trends observed.

Nomenclature describing BC has evolved alongside the methods we use to characterize this critical carbon fraction and definitions vary among scientific disciplines. For example, atmospheric communities describe BC as light-absorbing, soot-like aerosols, whereas soil scientists describe BC as the carbonaceous (carbon-rich) material that resists degradation in soils and sediments^19^. In the aquatic biogeochemistry community, pyrogenic carbon (PyC) refers to the entire continuum of molecules formed from fire and the term is often used to contextualize the condensed aromatic BC fraction. The term thermogenic carbon may also apply to BC, as it is used to describe organic matter that has been thermally altered by non-fire processes (e.g., geologic maturation, hydrothermal circulation). Since the use of specific scientific language promotes efficient communication within and among disciplines, we emphasize the use of clear and consistent definitions to describe BC. Thus, analytical windows must always be precisely articulated to avoid the confusion that results from propagation of poorly defined terminology. From an analytical perspective, it is also essential that the DBC community works together to establish consistency when reporting DBC-specific isotopic signatures. Specifically, a cross-laboratory comparison that incorporates multiple methods measuring BC reference materials^15^ is necessary to establish direct linkages between DBC reactivity and isotopic composition (Δ^14^C and δ^13^C). Clear and transparent corrections for radiocarbon data are also mandatory when reporting DBC Δ^14^C composition, because extensive processing of DBC data can drastically alter Δ^14^C values^9,20^. Since a universal method that covers the entire combustion continuum does not exist, we rely on a suite of methods to examine the complexity of thermally altered carbon, each capturing a different compositional and reactivity fraction (Fig. 1). By following the recommendations described here, we can best contribute to the global initiative to constrain DBC sources and fate along the land-to-ocean continuum.

## Data Availability

No datasets were generated for this manuscript.
